# Reference tissue normalization in longitudinal ^18^F-florbetapir positron emission tomography of late mild cognitive impairment

**DOI:** 10.1186/s13195-016-0172-3

**Published:** 2016-01-15

**Authors:** Sepideh Shokouhi, John W. Mckay, Suzanne L. Baker, Hakmook Kang, Aaron B. Brill, Harry E. Gwirtsman, William R. Riddle, Daniel O. Claassen, Baxter P. Rogers, for the Alzheimer’s Disease Neuroimaging Initiative

**Affiliations:** Department of Radiology and Radiological Sciences, Vanderbilt University Institute of Imaging Science, 1161 21st Avenue South, Medical Center North, AA-1105, Nashville, TN 37232-2310 USA; Center of Functional Imaging, Lawrence Berkeley National Laboratory, One Cyclotron Road, Berkeley, CA 94720 USA; Department of Biostatistics, Vanderbilt University, 2525 West End Avenue, 11th Floor, Suite 11000, Nashville, TN 37203-1738 USA; Department of Neurology, Vanderbilt University, A-0118 Medical Center North, 1161 21st Avenue South, Nashville, TN 37232-2551 USA; Department of Psychiatry, Vanderbilt University, 1601 23rd Avenue South, Nashville, TN 37212 USA

## Abstract

**Background:**

Semiquantitative methods such as the standardized uptake value ratio (SUVR) require normalization of the radiotracer activity to a reference tissue to monitor changes in the accumulation of amyloid-β (Aβ) plaques measured with positron emission tomography (PET). The objective of this study was to evaluate the effect of reference tissue normalization in a test–retest ^18^F-florbetapir SUVR study using cerebellar gray matter, white matter (two different segmentation masks), brainstem, and corpus callosum as reference regions.

**Methods:**

We calculated the correlation between ^18^F-florbetapir PET and concurrent cerebrospinal fluid (CSF) Aβ_1–42_ levels in a late mild cognitive impairment cohort with longitudinal PET and CSF data over the course of 2 years. In addition to conventional SUVR analysis using mean and median values of normalized brain radiotracer activity, we investigated a new image analysis technique—the weighted two-point correlation function (wS_2_)—to capture potentially more subtle changes in Aβ-PET data.

**Results:**

Compared with the SUVRs normalized to cerebellar gray matter, all cerebral-to-white matter normalization schemes resulted in a higher inverse correlation between PET and CSF Aβ_1–42_, while the brainstem normalization gave the best results (high and most stable correlation). Compared with the SUVR mean and median values, the wS_2_ values were associated with the lowest coefficient of variation and highest inverse correlation to CSF Aβ_1–42_ levels across all time points and reference regions, including the cerebellar gray matter.

**Conclusions:**

The selection of reference tissue for normalization and the choice of image analysis method can affect changes in cortical ^18^F-florbetapir uptake in longitudinal studies.

## Background

Amyloid-β (Aβ) plaques and neurofibrillary tau tangles are known pathological features of Alzheimer’s disease (AD) [1, 2] that manifest years before the onset of clinical symptoms [3–8]. Aβ plaques are identified in vivo using brain positron emission tomography (PET) with several radiotracers, including ^11^C-Pittsburgh Compound B (^11^C-PiB) [9], ^18^F-florbetapir [10], ^18^F-FDDNP [11], ^18^F-florbetaben [12], and ^18^F-flutemetamol [13]. The standardized uptake value ratio (SUVR) is a semiquantitative method frequently used in clinical trials of antiamyloid drugs to monitor the accumulation and progression of Aβ plaques and to assess the effects of antiamyloid drug therapy. The SUVR method is used in most large studies because it is easily calculated and does not require long dynamic scans or measurement of the arterial input function. Nevertheless, it requires normalization of regional PET activity to a reference tissue to account for nonspecific radiotracer binding. Because ^11^C-PiB and ^18^F-florbetapir target predominately the classic core and neuritic Aβ plaques, which are not evidenced in the cerebellum [14–17], whole cerebellum (or the cerebellar gray matter) is commonly used as a reference region [18, 19]. However, recent research raises new concerns about the accuracy of the SUVR measures using cerebellar normalization. In particular, the variability observed in the longitudinal progression of SUVR values seems to be discrepant with the expected values on the basis of pathological and biological grounds.

In recent studies [20–23], researchers have examined the feasibility of alternative reference regions for amyloid-PET. Brendel and colleagues [20] used the discriminatory power between AD, mild cognitive impairment (MCI), and healthy control (HC) subject groups, as well as the magnitude and variability of temporal changes in ^18^F-florbetapir PET, to evaluate different reference tissue. Chen and colleagues [21] examined the strength of associations between ^18^F-florbetapir PET increase and clinical decline in addition to means of tracking the magnitude and variability of longitudinal Aβ-PET changes in different subject groups. Landau and colleagues [22] stratified the following subject groups on the basis of their cerebrospinal fluid (CSF) Aβ_1–42_ levels at baseline: (1) a control group that included healthy subjects with normal and stable CSF Aβ_1–42_ levels and (2) a second group that included both cognitively healthy subjects and those with early amnestic MCI with abnormal CSF Aβ_1–42_ levels at baseline. The study was designed to test if the cortical Aβ-PET levels in the HC group remained stable while they increased in the second group. All three of these studies incorporated static ^18^F-florbetapir PET scans (summarized in Table 1). In another study, by Wong and colleagues [23], the distribution volume ratio in a dynamic ^18^F-FDDNP PET scan was used to determine the discriminatory power between an HC group and the AD group. In all of these studies, researchers found that use of white matter normalization improved the accuracy of longitudinal Aβ-PET data more strongly than use of gray matter normalization.

**Table 1:** Summary of previous longitudinal ^18^F-florbetapir PET studies for comparison between reference tissues for normalization of PET activity

	Brendel et al. [20]	Chen et al. [21]	Landau et al. [22]
Reference regions	Whole cerebellum	Whole cerebellum	Cerebellar gray matter
	Brainstem	Pons	Whole cerebellum
	White matter	White matter	White matter
			Brainstem/pons
			Composite ROI
Subject groups	MCI (*n* = 483)	MCI (*n* = 187)	CSF- (14)
	AD (*n* = 163)	AD (*n* = 31)	CSF+ (*n* = 91)
	HC (*n* = 316)	HC (*n* = 114)	
PET radiotracer	^18^F-florbetapir	^18^F-florbetapir	^18^F-florbetapir
Image analysis	Mean SUV/SUVR	Mean SUVR	Mean SUVR
Evaluation method	Discrimination power between subject groups, variability in longitudinal increase in Aβ-PET	Longitudinal increase in Aβ-PET and association with clinical decline	Physiological plausible longitudinal increase in Aβ-PET
Best reference region	White matter/brainstem with partial volume correction	White matter	Reference regions containing white matter

*Aβ* amyloid-β, *AD* Alzheimer’s disease, *CSF* cerebrospinal fluid, *HC* healthy controls, *MCI* mild cognitive impairment, *PET* positron emission tomography, *ROI* region of interest, *SUV* standardized uptake value, *SUVR* standardized uptake value ratio

The objective of our present work was to complement the previous research by the use of a new PET image analytical method as well as longitudinal data of both CSF Aβ_1–42_ levels and ^18^F-florbetapir images to identify which reference region normalization results in the optimal visit-to-visit correlation between these two biomarkers of AD pathology. The subjects in this study were those diagnosed with late mild cognitive impairment (LMCI) from the ADNI 2 phase of the Alzheimer’s Disease Neuroimaging Initiative (ADNI) with stable CSF Aβ_1–42_ levels at baseline and at 24-month follow-up; thus, longitudinal changes in ^18^F-florbetapir PET were not expected to occur, which allows use of their PET images as a test–retest dataset to evaluate the effect of reference region normalization. All PET images are analyzed with the conventional SUVR mean and median measures and with a new PET image cluster analysis tool based on a weighted two-point correlation (wS_2_). The wS_2_ method is a statistical tool adopted from astronomy and materials science and can be used to detect specific changes in spatial patterns within Aβ-PET images that we refer to as *increased clustering* or *flocculence*. Our preliminary data [24] indicate the potential utility of this method for detecting longitudinal changes that are difficult to assess with conventional regional mean image values, which typically have large standard deviations.

## Methods

### Alzheimer’s Disease Neuroimaging Initiative

Data used in the preparation of this article were obtained from the ADNI database (adni.loni.usc.edu). The ADNI was launched in 2003 as a public-private partnership led by Principal Investigator Michael W. Weiner, MD. The primary goal of ADNI has been to test whether serial magnetic resonance imaging (MRI), PET, other biological markers, and clinical and neuropsychological assessments can be combined to measure the progression of MCI and early AD.

### Subject selection

Data from 21 ADNI subjects with LMCI were used in our study. We included all subjects with LMCI who had ^18^F-florbetapir PET and T1-weighted MRI images at baseline and 24-month follow-up scans following the PET technical procedures of the ADNI 2 phase. We further limited our subject selection to patients with LMCI who had longitudinal CSF data obtained at time points close to their PET baseline and follow-up scans. The specific selection of the LMCI subject group from the ADNI 2 phase was based on their stable levels of CSF Aβ_1–42_, which allowed use of their corresponding longitudinal PET images as a test–retest dataset. While our selection criteria limited the number of available subjects, one of the main advantages of using the ADNI 2 data was the commonality of the image acquisition protocols, which ensured consistency of data within and between sites and thus reduced heterogeneity that would have otherwise added to the variability of both longitudinal and cross-sectional data. The biomarker datasheet containing the CSF Aβ_1–42_ levels was downloaded from the ADNI archive. The dataset is named UPENN–CSF Biomarkers [ADNI GO/2] version 2013-10-31.

**Fig. 1:**
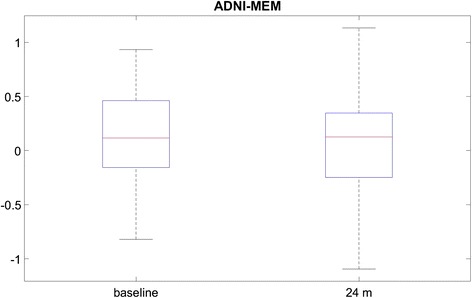
Box plots of the Alzheimer’s Disease Neuroimaging Initiative composite memory score (ADNI-MEM), combining the Rey Auditory Verbal Learning Test, the Logical Memory Test of the Wechsler Memory Scale, the Mini Mental State Examination and the Alzheimer’s Disease Assessment Scale–Cognitive subscale

Table 2 summarizes the demographic information of the subjects enrolled in this study. Both the baseline and follow-up Aβ_1–42_ CSF values (measured as picograms per milliliter) matched the average ADNI values of the MCI cohort (baseline 165 ± 45 pg/ml, 24 months 161 ± 46 pg/ml). There was no significant change in CSF Aβ_1–42_ levels between baseline and follow-up among these subjects. This was determined on the basis of the coefficient of variation of CSF values between the two time points, which was on average 3.34 % across our cohort. For comparison, the longitudinal within-laboratory coefficient of variation for CSF measures is typically 5–19 % [25]. In addition to the CSF values, our subjects’ cognitive test scores, measured using the Alzheimer’s Disease Assessment Scale–Cognitive subscale (ADAS-cog) [26], were 18 ± 7 at baseline and 19 ± 10 at follow-up. The Clinical Dementia Rating scores at both baseline and 24 months were 0.5 for almost all subjects. The Mini Mental State Examination (MMSE) [27] scores were 28 ± 2 at baseline and 26 ± 3 at follow-up. To summarize the subjects’ clinical status, we included the box plots of their ADNI composite memory score [28], which combines the Rey Auditory Verbal Learning Test, the Logical Memory Test of the Wechsler Memory Scale, the MMSE, and the ADAS-cog (Fig. 1).

**Table 2:** Clinical and demographic data of the ADNI subjects in this study

Parameter	Data
Subjects, *n*	20
Females, *n*	11
Baseline age, yr	73 ± 8
APOE A1/A2 carriers, *n*	9
Time between PET scan and CSF, days	6 ± 14
CSF Aβ_1–42_, pg/ml	165 ± 45 (baseline) and 161 ± 46 (follow-up)
ADAS-cog score	18 ± 7 (baseline) and 19 ± 10 (follow-up)
Clinical Dementia Rating score	0.5 ± 0 (baseline) and 0.5 ± 0.3 (follow-up)
Mini Mental State Examination score	28 ± 2 (baseline) and 26 ± 3 (follow-up)

Aβ, Amyloid-β; ADAS-cog, Alzheimer’s Disease Assessment Scale–Cognitive subscale; ADNI, Alzheimer’s Disease Neuroimaging Initiative; APOE, apolipoprotein E; CSF, cerebrospinal fluid; PET, positron emission tomography. Data type in this table are number, age, days, CSF levels and results of the test scores

### Data acquisition, image reconstruction, and preprocessing

All patient data were acquired at participating ADNI sites. ^18^F-florbetapir PET, together with concurrent T1-weighted MRI volumes at baseline and 24-months follow-up, were downloaded from the ADNI database. The detailed description of the acquisition protocol can also be found on the ADNI website (http://adni.loni.usc.edu/). According to the ADNI protocols, a 370-MBq bolus injection of radiotracer was administered. This was followed by a 20-minute continuous brain PET imaging session that began approximately 50 minutes after the injection. The images were reconstructed immediately after the 20-minute scan according to scanner-specific reconstruction protocols, each using different versions of a maximum likelihood algorithm, to assess the scan quality and potential presence of motion artifacts. All images were corrected for attenuation and scatter according to the scanner-specific protocols. Upon completion, the imaging data were uploaded to the data archive of the Laboratory of Neuro Imaging at the University of Southern California, where they were coregistered and averaged. These are the datasets used in this study.

### Image analysis

**Fig. 2:**
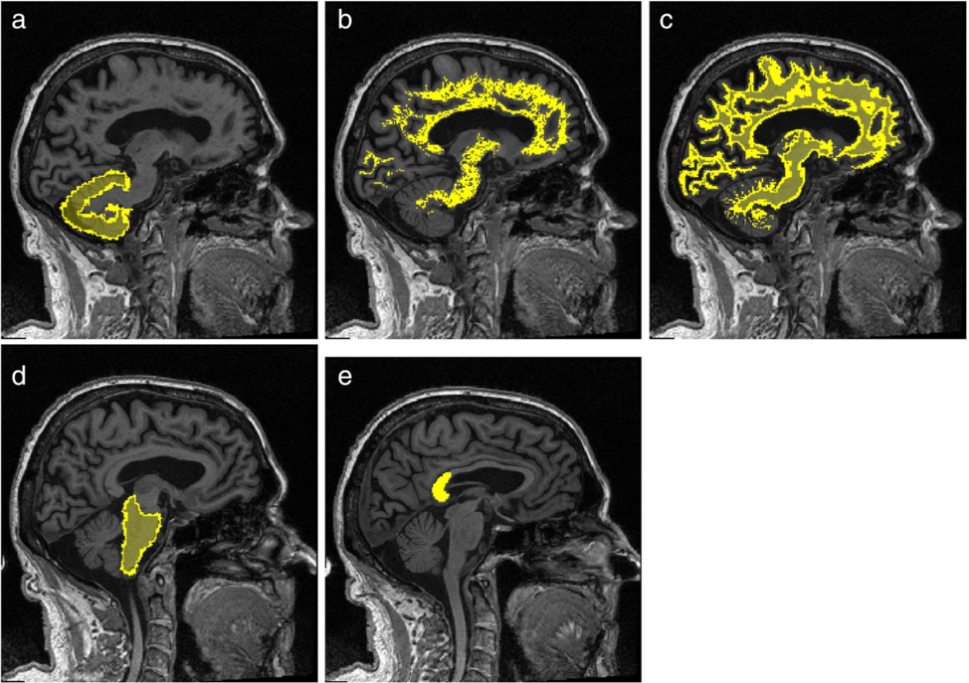
Reference tissue masks. Cerebellar gray matter (**a**), 100 % threshold white matter mask (**b**), 10 % threshold white matter mask (**c**) , brainstem (**d**), and splenium of corpus callosum (**e**)

^18^F-florbetapir images of each subject were aligned to their concurrent T1-weighted MRI volume. Gray matter and white matter masks of the T1-weighted MRI volumes were segmented in each subject’s native space using SPM12 software (Wellcome Trust Centre for Neuroimaging, London, UK). Two different thresholds were applied on the segmented white matter to generate two types of white matter masks. The 10 % white matter mask included white matter voxels that were adjacent to gray matter. These border voxels were removed in the 100 % white matter mask. The template-based regional masks from the cerebellar gray matter and brainstem were obtained from the SPM12 atlas (labels_Neuromorphometrics.nii) and deformed into the subject’s native space. Regional masks for the corpus callosum were drawn manually. This was done by importing the MRI volumes into Amide, a medical image display and data analysis tool [29], where the center slice of the sagittal view was used to draw a region of interest around the splenium of the corpus callosum. Figure 2 represents candidate reference regions overlaid on a subject’s T1-weighted MRI scan. The cerebral brain gray matter PET signal was normalized with respect to each mask, and the SUVR mean and median values were calculated.

In addition to the SUVR mean and median values, we also calculated the wS_2_ of the florbetapir PET images. The wS_2_ method is a statistical image analytical method commonly used in astronomy [30] and materials science [31]. With this method, we derived a quantitative parameter from PET images to characterize the heterogeneity of the Aβ-PET activity distribution, which we refer to as the *clustering or flocculence*. The wS_2_ analysis was also implemented with normalized Aβ-PET images. However, unlike the regional mean and median values, changes in wS_2_ more specifically reflect changes in the spatial patterns of activity. Thus, these changes are potentially less sensitive to minor temporal variations in the reference tissue activity (variations in normalization threshold). PET analysis using the wS_2_ method also results in smaller standard errors and thus may be more suitable for detecting subtle changes due to the larger effect size. The theoretical framework of wS_2_ is described in our previous work where this method was validated with ^11^C-PiB PET data [24].

**Fig. 3:**
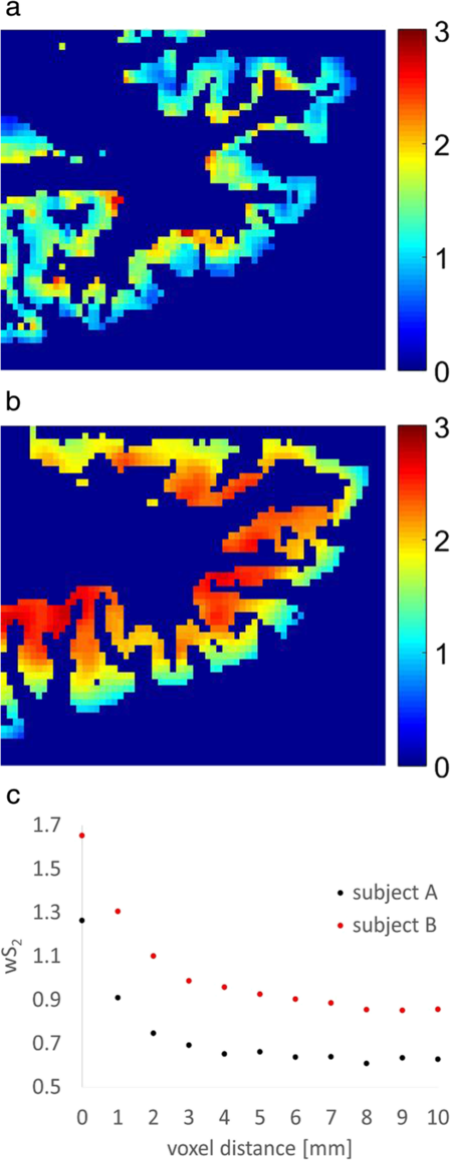
^18^F-florbetapir positron emission tomographic images (zoomed over an axial slice located in the frontal lobe) from two subjects (**a**) with low tracer uptake and (**b**) with high tracer uptake, as well as (**c**) the weighted two-point correlation function (wS_2_) calculated from whole-brain images of these two subjects

The calculation of wS_2_ started with sampling 50,000 random voxel pairs located within the gray matter of the ^18^F-florbetapir PET image volume. For each voxel pair (each sampling instance), a weighting factor was calculated as the product of two terms. The first term was the average value of the two voxels, and the second term incorporated the absolute difference between the two voxel values into an exponential term. The weighting factor of an instance is higher when the values of both voxels are high and these values are close to each other. All sampling instances were then binned by the intervoxel distances, and for a given distance *r* the sum of the weighting factors was divided by the total number of instances with distance *r* and plotted versus *r* to obtain a wS_2_ between 0 and 10 mm. Both the slope and the wS_2_ area under the curve (AUC) change with the increased activity and increased heterogeneity of the activity distribution within the brain. Figure 3 shows the wS_2_ AUCs from two florbetapir PET images. The wS_2_ AUC was used as the quantitative outcome of this analysis and was calculated together with the mean and median of the SUVR for all baseline and follow-up images. The coefficients of variation of SUVR mean and median, as well as wS_2_ across different time points and normalization schemes, were calculated over all 21 participants. Spearman’s rank correlation coefficient was calculated between the ^18^F-florbetapir PET outcomes (SUVR mean and median and ws_2_) and the CSF Aβ_1–42_ at baseline and follow-up.

## Results

**Fig. 4:**
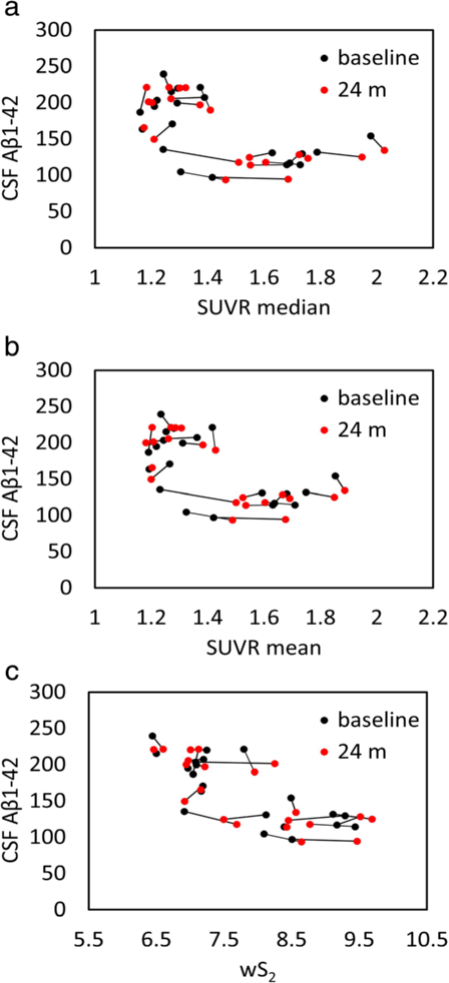
Scatterplots of all cerebrospinal fluid (CSF) amyloid-β_1–42_ (Aβ_1–42_) versus standardized uptake value ratio (SUVR) median (**a**), mean (**b**), and weighted two-point correlation function (wS_2_) (**c**) values obtained by normalization of positron emission tomography activity to cerebellar gray matter at baseline (*black dots*) and 24-month follow-up (*red dots*)

**Fig. 5:**
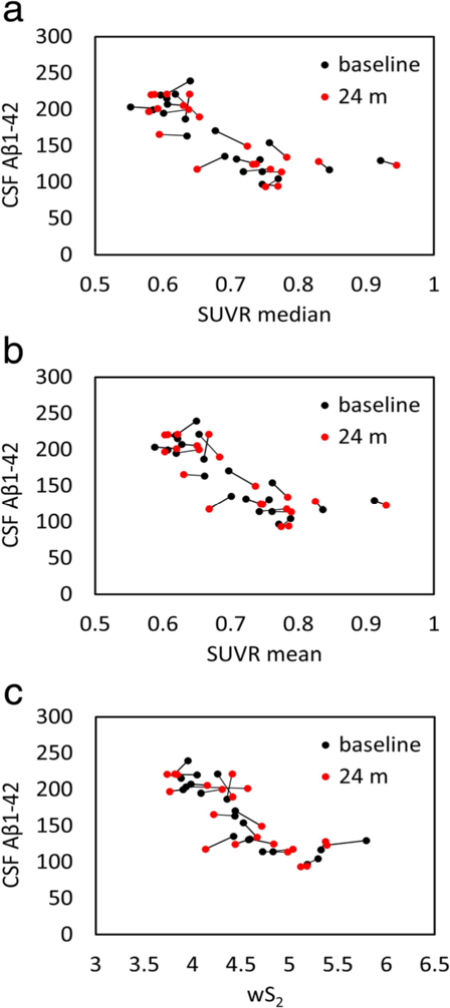
Scatterplots of all cerebrospinal fluid (CSF) amyloid-β_1–42_ (Aβ_1–42_) versus standardized uptake value ratio (SUVR) median (**a**), mean (**b**), and weighted two-point correlation function (wS_2_) (**c**) values obtained by normalization of positron emission tomography activity to white matter (10 %) at baseline (*black dots*) and 24-month follow-up (*red dots*)

**Fig. 6:**
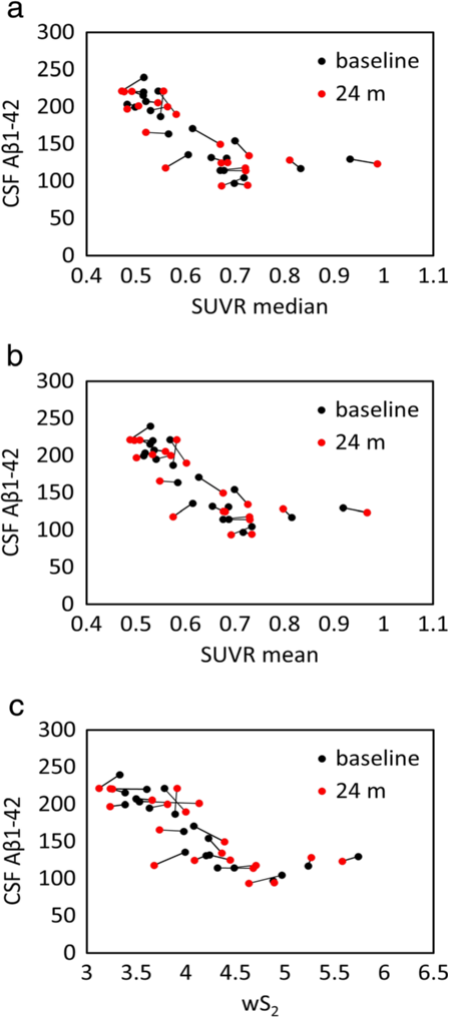
Scatterplots of all cerebrospinal fluid (CSF) amyloid-β_1–42_ (Aβ_1–42_) versus standardized uptake value ratio (SUVR) median (**a**), mean (**b**), and weighted two-point correlation function (wS_2_) (**c**) values obtained by normalization of positron emission tomography activity to white matter (100 %) at baseline (*black dots*) and 24-month follow-up (*red dots*)

**Fig. 7:**
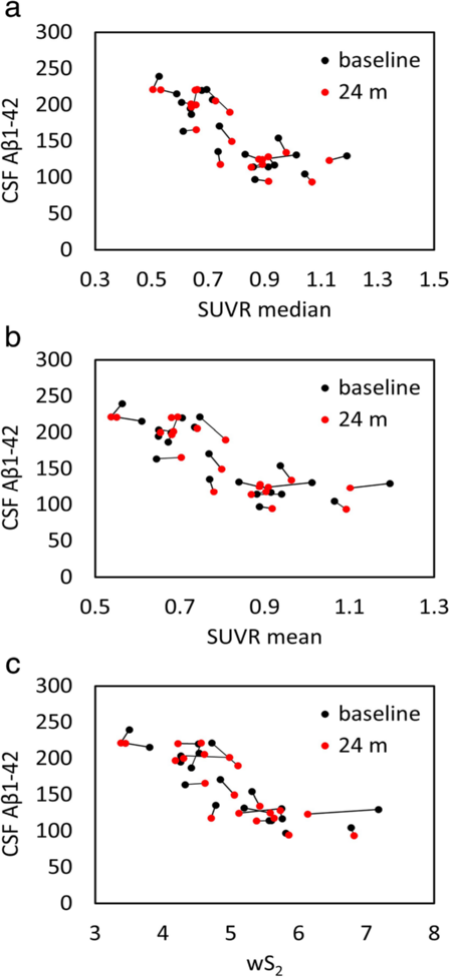
Scatterplots of all cerebrospinal fluid (CSF) amyloid-β_1–42_ (Aβ_1–42_) versus standardized uptake value ratio (SUVR) median (**a**), mean (**b**), and weighted two-point correlation function (wS_2_) (**c**) values obtained by normalization of positron emission tomography activity to brainstem at baseline (*black dots*) and 24 months follow-up (*red dots*)

**Fig. 8:**
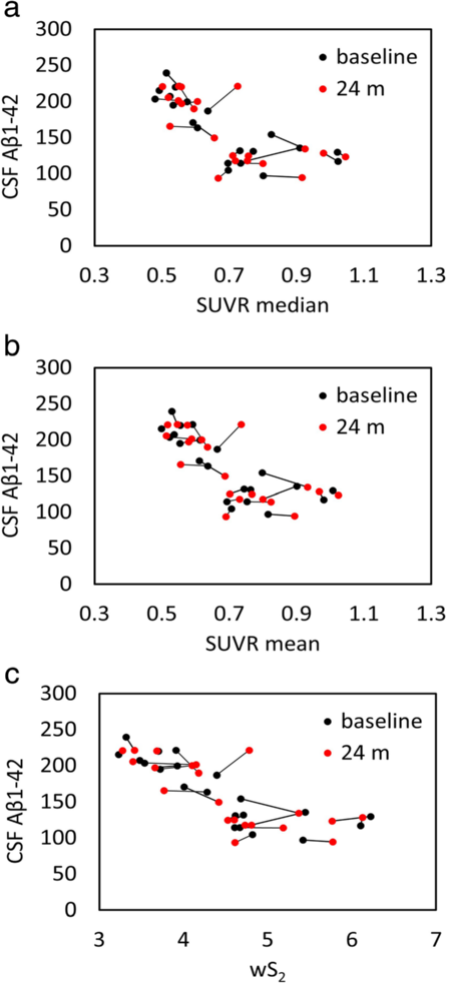
Scatterplots of all cerebrospinal fluid (CSF) amyloid-β_1–42_ (Aβ_1–42_) versus standardized uptake value ratio (SUVR) median (**a**), mean (**b**), and weighted two-point correlation function (wS_2_) (**c**) values obtained by normalization of positron emission tomography activity to corpus callosum at baseline (*black dots*) and 24-month follow-up (*red dots*)

**Fig. 9:**
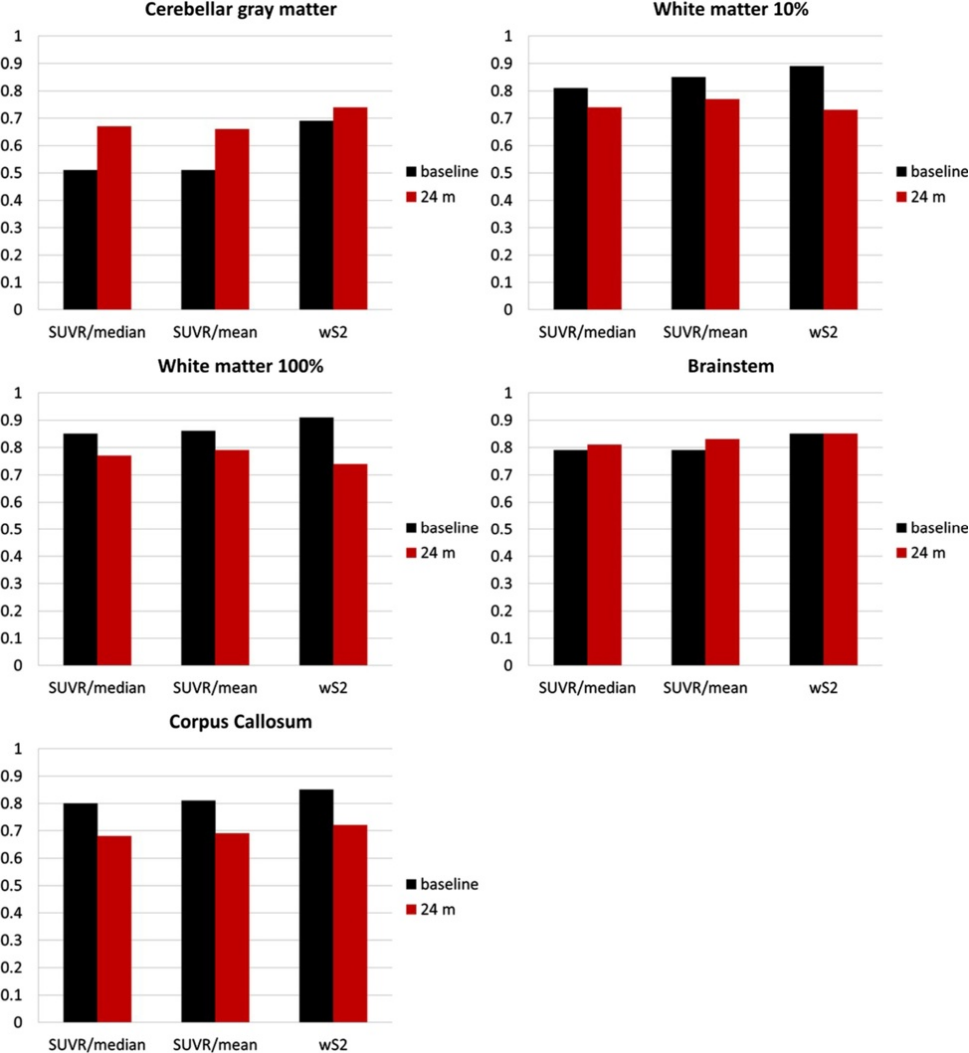
Spearman’s rank correlation between cerebrospinal fluid (CSF) amyloid-β_1–42_ (Aβ_1–42_) and ^18^F-florbetapir standardized uptake value ratio (SUVR) median and mean and weighted two-point correlation function (wS_2_) measures at baseline (*black bars*) and 24-month follow-up (*red bars*) for five different reference tissue normalization schemes

We used five different normalization regions (Fig. 2) to evaluate the correlation between amyloid-PET and CSF measures in a test–retest study. This association is graphically illustrated for all subjects at baseline and follow-up in Fig. 4 (cerebellar gray matter), Fig. 5 (10 % white matter), Fig. 6 (100 % white matter), Fig. 7 (brainstem), and Fig. 8 (corpus callosum). The medium and mean SUVR values and the wS_2_ AUC were plotted (*x*-axis) versus the CSF Aβ_1–42_ (*y*-axis). For each subject, the baseline marker (black) was connected via a line to the follow-up marker (red) to show each subject’s individual change. Qualitatively, the scatterplot of SUVR mean and median values versus CSF Aβ_1–42_ showed the lowest linear association between the two biomarkers when the cerebellar gray matter was selected as the reference region (Fig. 4a and b). With cerebellar normalization, the global mean and median SUVR values were between 1.1 and 2.0. The CSF Aβ_1–42_ of brains with mean and median SUVR less than 1.5 seemed to remain clustered around 200 pg/ml, whereas SUVR mean and median values greater than 1.5 were associated with CSF Aβ_1–42_ values around 125 pg/ml. The scatterplots of the wS_2_ outcomes showed a more linear association with CSF Aβ_1–42_ for all normalization schemes including the cerebellar gray matter (Fig. 4.C). This association was quantitatively evaluated by using Spearman’s rank correlation coefficient (Fig. 9, Table 3) between the two biomarkers at both baseline (black bar) and follow-up (red bar). While the correlation was statistically significant for all normalization schemes, time points and methods of analysis, it was modest (~0.5) when cerebellar gray matter was selected as reference tissue and the SUVR mean and median values were calculated for PET analysis. The brainstem normalization resulted in the highest and most stable (lowest variability) Spearman’s rank correlation values (~0.8) across both time points and all three methods of analysis. The coefficient of variation across all time points and normalization schemes was 0.10 for wS_2_ method, 0.14 for SUVR mean and 0.13 for SUVR median.

**Table 3:** Spearman’s rank correlation between CSF Aβ_1–42_ and PET measures

	Baseline			Follow-up		
	SUVR median	SUVR mean	wS_2_	SUVR median	SUVR mean	wS_2_
Cerebellum	0.51	0.51	0.69	0.67	0.66	0.74
White matter (10 %)	0.81	0.85	0.89	0.74	0.77	0.73
White matter (100 %)	0.85	0.86	0.91	0.77	0.79	0.74
Brainstem	0.79	0.79	0.85	0.81	0.83	0.85
Corpus callosum	0.8	0.81	0.85	0.68	0.69	0.72

*SUVR* standardized uptake value ratio; *wS*_*2*_ weighted two-point correlation function

## Discussion

The results of this study show that analysis of ^18^F-florbetapir PET data normalized to white matter reference regions results in a higher inverse correlation to CSF Aβ_1–42_ and that this correlation exhibits less variability over time compared with ^18^F-florbetapir PET data that are normalized to cerebellar gray matter (Table 3, Fig. 9). These findings are in agreement with recent studies [20–23] in which researchers investigated the effect of reference tissue normalization using a significantly larger number of ADNI subjects. This good agreement despite a smaller cohort in our study could be partially attributed to our subject selection, which consisted of ADNI2 patients with LMCI. As described in the Methods section, the image acquisition protocols of ADNI 2 were designed to ensure consistency of data within and between sites. All our subjects had ^18^F-florbetapir PET scans at baseline and 24-month follow-up using the same (within-subject) scanner and the same image reconstruction and correction methods. These factors may have helped to reduce potential heterogeneities within this cohort that would otherwise have added to variability in both longitudinal and cross-sectional data.

Another advantage of ADNI 2 is the availability of concurrent CSF Aβ_1–42_ at both baseline and 24-month follow-up time points, which allowed us to use them as a reference method to correlate with PET data at two different time points. On the basis of their stable CSF Aβ_1–42_, the brain amyloid levels of these subjects were not expected to change between baseline and the 24-month follow-up PET scans, thus making the ^18^F-florbetapir PET images from this cohort an appropriate dataset for test–retest variability assessment of reference region normalization. The observed stable CSF Aβ_1–42_ was not unexpected for subjects with LMCI, because it is known that the biomarkers of amyloid deposition approach a plateau by the onset time of LMCI and clinical AD [30].

Using a cohort with stable CSF Aβ_1–42_, our objective was to find a reference tissue that would give the highest and most stability (lowest variability) in Spearman’s rank correlation between these two biomarkers calculated at two time points. While all white matter–normalized SUVRs indicated higher correlation to CSF measures than the cerebellar normalization, the brainstem normalization gave the best results among the white matter regions despite its location at the edge of the PET scanner field of view (FOV). The location of the cerebellum was suspected to be the main reason for variability observed in the previous studies [21, 22]. Due to their location, both brainstem and cerebellum are subject to increased scatter and decreased geometric sensitivity. However, PET data undergo rigorous attenuation, scatter, and normalization corrections to ensure uniformity within the FOV. Also, given that in our study the correlation values for cerebellar normalization were at their lowest levels for both baseline and follow-up time points, other factors, such as biological effects, could be more relevant than scanner-related physical effects. The connecting lines in Fig. 4 show that the within-subject differences between baseline and follow-up PET data (mainly intermediate SUVR mean and median values) were larger than all other normalization schemes (Figs. 5, 6, 7 and 8). In these figures, it is also apparent that the association between all three PET analytical methods and CSF measures become increasingly nonlinear as the PET values increase. This nonlinearity effect was most prominent when cerebellar gray matter was used as reference tissue (Fig. 4), where the CSF data of SUVR mean and median values below 1.5 were clustered around 200 pg/ml and the CSF data of SUVR mean and median values above 1.5 corresponded to CSF measures that remained around 125 pg/ml. All other white matter normalization schemes resulted in slightly more linear associations with CSF measures, in particular for intermediate PET values.

We included CSF because Aβ accumulation has been hypothesized to result from an imbalance between Aβ production and clearance [2, 32–35]. In particular, the impairment of clearance mechanisms seems to be the main cause of Aβ accumulation in sporadic or late-onset forms of AD [35], which account for the majority of patients with AD. In several previous studies, researchers have observed a relationship between cortical amyloid tracer binding and levels of CSF Aβ_1–42_ using ^11^C-PiB [36] and ^18^F-florbetapir [37]. These studies, which were based on cerebellar normalization, showed that the CSF levels decreased with increased radiotracer uptake but reached a plateau at higher SUVR values. We made a similar observation with cerebellum normalization (Fig. 4a and b). Other reference region normalizations, the brainstem in particular, resulted in more linear relationships across a wide range of cortical radiotracer uptake values at both baseline and follow-up. We emphasize on the importance of this observation because the axial location of the cerebellum (increased scatter and attenuation) accounted for the observed longitudinal variabilities in previous studies. However, scanner-related effects would affect the PET–CSF association within the whole spectrum of SUVR values. Also, both the brainstem and the cerebellum are equally subject to increased scatter and decreased geometric sensitivity. Our approach might indicate that the variability associated with the reference region normalization may more likely be related to biological factors than to scanner-related effects.

Four different white matter masks (white matter 10 %, white matter 100 %, brainstem, and splenium of corpus callosum) were applied. While the white matter 10 % included the white matter regions that shared borders with gray matter, these regions were removed in the 100 % white matter mask. Correlation values from these two white matter masks and the corpus callosum were similar.

The wS_2_ technique was used as an additional method complementary to the conventional SUVR analysis that is performed by calculating regional mean and median SUVR values. Compared with the SUVR mean and median values, the wS_2_ metric was associated with the highest average Spearman’s rank correlation across all time points and reference regions, including the cerebellar gray matter. Given that the wS_2_ metric is based on changes in image spatial patterns, we expected that this method would be slightly less sensitive to minor temporal variations in reference region radiotracer activity, which would cause variations in normalization thresholds. The wS_2_ method evaluates associations between voxel values at different distances. These associations remain preserved, to some extent, even when the normalization threshold varies.

To date, we have applied the wS_2_ analysis with two different radiotracers (^11^C-PiB and ^18^F-florbetapir) and have been able to show consistent results. Using a statistical analysis, we evaluated the effect of injected dose (as a surrogate for image noise) and the region size on the wS_2_ outcomes and made a comparison with SUVR mean and median values. We obtained high and stable correlations between CSF Aβ levels and wS_2_ outcomes with both radiotracers. Further validations would require a full quantitative analysis using kinetic modeling and dynamic acquisitions. Our main future objective is to test the wS_2_ methodology with dynamic PET scans and list-mode data acquisition to investigate how different image acquisition (starting time point and duration) and reconstruction parameters (number of iterations and noise regularization) can change the image spatial patterns and subsequently the wS_2_ outcomes. Image preprocessing is another important factor. Spatial resolutions of human PET scanners range from greater than 2.5-mm full-width half-maximum (FWHM) in some research scanners to greater than 7-mm FWHM in many commonly used clinical PET systems [38–40]. Additional preprocessing steps, such as image smoothing, further reduce the image resolution from 7- to 12-mm FWHM. For example, most reported ADNI analyses use level 4 preprocessed imaging data, which are smoothed to a uniform isotropic resolution of 8-mm FWHM [39]. The smoothing process is beneficial for cross-sectional comparisons and for qualitative visual reads by clinicians, due to the improved uniformity. However, it has a disadvantage in that potentially important high-resolution spatial patterns are smoothed away [40]. The spatial smoothing of within-subject longitudinal can reduce the effect size [41]. We are the first group, to our knowledge, to propose a method designed to improve understanding of the nature of nonuniform spatial activity patterns that explain the impact of spatial smoothing on longitudinal changes.

## Conclusions

The selection of reference tissue for normalization of ^18^F-florbetapir PET images as well as the image analysis method can modify the quantitative outcomes in longitudinal studies. Understanding factors that contribute to temporal variations of reference region radiotracer uptake merits further investigation.

## Abbreviations

Aβ: amyloid-β
AD: Alzheimer’s disease
ADAS-cog: Alzheimer’s Disease Assessment Scale–Cognitive subscale
ADNI: Alzheimer’s Disease Neuroimaging Initiative
ADNI-MEM: Alzheimer’s Disease Neuroimaging Initiative composite memory score
APOE: apolipoprotein E
AUC: area under the curve
CSF: cerebrospinal fluid
^11^C-PiB: ^11^C-Pittsburgh Compound B
FOV: field of view
FWHM: full-width half-maximum
HC: healthy control
LMCI: late mild cognitive impairment
MCI: mild cognitive impairment
MMSE: Mini Mental State Examination
MRI: magnetic resonance imaging
PET: positron emission tomography
ROI: region of interest
SUV: standardized uptake value
SUVR: standardized uptake value ratio
wS_2_: weighted two-point correlation function
